# A deep‐learning workflow to predict upper tract urothelial carcinoma protein‐based subtypes from H&E slides supporting the prioritization of patients for molecular testing

**DOI:** 10.1002/2056-4538.12369

**Published:** 2024-03-19

**Authors:** Miriam Angeloni, Thomas van Doeveren, Sebastian Lindner, Patrick Volland, Jorina Schmelmer, Sebastian Foersch, Christian Matek, Robert Stoehr, Carol I Geppert, Hendrik Heers, Sven Wach, Helge Taubert, Danijel Sikic, Bernd Wullich, Geert JLH van Leenders, Vasily Zaburdaev, Markus Eckstein, Arndt Hartmann, Joost L Boormans, Fulvia Ferrazzi, Veronika Bahlinger

**Affiliations:** ^1^ Institute of Pathology, University Hospital Erlangen‐Nürnberg, Friedrich‐Alexander‐Universität Erlangen‐Nürnberg (FAU) Erlangen Germany; ^2^ Comprehensive Cancer Center Erlangen‐EMN (CCC ER‐EMN) Erlangen Germany; ^3^ Bavarian Cancer Research Center (BZKF) Erlangen Germany; ^4^ Department of Urology Erasmus MC Urothelial Cancer Research Group Rotterdam The Netherlands; ^5^ Institute of Pathology, University Medical Center Mainz Mainz Germany; ^6^ Department of Urology Philipps‐Universität Marburg Marburg Germany; ^7^ Department of Urology and Pediatric Urology University Hospital Erlangen, Friedrich‐Alexander Universität Erlangen‐Nürnberg (FAU) Erlangen Germany; ^8^ Department of Pathology Erasmus MC Cancer Institute, University Medical Centre Rotterdam the Netherlands; ^9^ Department of Biology Friedrich‐Alexander‐Universität Erlangen‐Nürnberg (FAU) Erlangen Germany; ^10^ Max‐Planck‐Zentrum für Physik und Medizin Erlangen Germany; ^11^ Department of Nephropathology Institute of Pathology, University Hospital Erlangen‐Nürnberg, Friedrich‐Alexander‐Universität Erlangen‐Nürnberg (FAU) Erlangen Germany; ^12^ Department of Pathology and Neuropathology University Hospital and Comprehensive Cancer Center Tübingen Tübingen Germany

**Keywords:** deep‐learning, digital pathology, immunohistochemistry, protein‐based subtypes, targeted therapy, upper tract urothelial carcinoma, whole slide images

## Abstract

Upper tract urothelial carcinoma (UTUC) is a rare and aggressive, yet understudied, urothelial carcinoma (UC). The more frequent UC of the bladder comprises several molecular subtypes, associated with different targeted therapies and overlapping with protein‐based subtypes. However, if and how these findings extend to UTUC remains unclear. Artificial intelligence‐based approaches could help elucidate UTUC's biology and extend access to targeted treatments to a wider patient audience. Here, UTUC protein‐based subtypes were identified, and a deep‐learning (DL) workflow was developed to predict them directly from routine histopathological H&E slides. Protein‐based subtypes in a retrospective cohort of 163 invasive tumors were assigned by hierarchical clustering of the immunohistochemical expression of three luminal (FOXA1, GATA3, and CK20) and three basal (CD44, CK5, and CK14) markers. Cluster analysis identified distinctive luminal (*N* = 80) and basal (*N* = 42) subtypes. The luminal subtype mostly included pushing, papillary tumors, whereas the basal subtype diffusely infiltrating, non‐papillary tumors. DL model building relied on a transfer‐learning approach by fine‐tuning a pre‐trained ResNet50. Classification performance was measured via three‐fold repeated cross‐validation. A mean area under the receiver operating characteristic curve of 0.83 (95% CI: 0.67–0.99), 0.8 (95% CI: 0.62–0.99), and 0.81 (95% CI: 0.65–0.96) was reached in the three repetitions. High‐confidence DL‐based predicted subtypes showed significant associations (*p* < 0.001) with morphological features, i.e. tumor type, histological subtypes, and infiltration type. Furthermore, a significant association was found with programmed cell death ligand 1 (PD‐L1) combined positive score (*p* < 0.001) and *FGFR3* mutational status (*p* = 0.002), with high‐confidence basal predictions containing a higher proportion of PD‐L1 positive samples and high‐confidence luminal predictions a higher proportion of *FGFR3*‐mutated samples. Testing of the DL model on an independent cohort highlighted the importance to accommodate histological subtypes. Taken together, our DL workflow can predict protein‐based UTUC subtypes, associated with the presence of targetable alterations, directly from H&E slides.

## Introduction

Urothelial carcinomas (UCs) are malignant epithelial neoplasms arising from the urothelial lining of the urinary tract [1]. The rare upper tract UC (UTUC) represents 5–10% of all UCs, whereas the remaining 90–95% are urothelial bladder cancer (UBC). UTUC is frequently associated with poor prognosis, with two‐thirds of patients being diagnosed at an invasive tumor stage [2]. Owing to the histopathological similarity between UTUC and UBC [3, 4], and the preponderance of the latter, UTUC is an understudied disease. However, a better understanding of UTUC biology could allow the identification of distinctive molecular traits with potential strong impact on patient stratification and treatment [3, 4, 5].

In recent years, transcriptome‐based subtyping allowed an improved stratification of several cancer entities into subgroups of patients sharing similar molecular features [6]. In muscle‐invasive BC (MIBC), different molecular subtypes have been proposed [7, 8, 9, 10, 11, 12], and in 2020 a consensus classification identified luminal and basal as the two distinctive subtypes [1, 13]. These subtypes offer valuable support for guiding targeted therapy options. Indeed, the luminal subtype appears associated with higher responsiveness to fibroblast growth factor receptor 3 (*FGFR3*)‐targeted therapies, and the basal subtype to immunotherapies such as programmed cell death ligand 1 (PD‐L1) and programmed cell death protein 1 (PD‐1) inhibitors [13, 14]. For UTUC, only few studies have so far investigated its genomic and transcriptomics landscape [15, 16, 17]. Additionally, no consensus subtypes have been identified yet [2, 3].

Assessment of molecular subtypes via high‐throughput sequencing is neither ubiquitously available nor cost‐effective. Thus, alternative subtyping approaches should be considered. In MIBC, studies showed a substantial overlap between molecular and protein‐based subtypes [18, 19], identifiable via more widespread, routinely applicable immunohistochemistry (IHC) analyses. Moreover, AI‐based approaches have recently emerged as novel research tools able to provide automated and accurate pathological diagnoses, leveraging the information residing into whole slide images (WSIs) [20]. Here, we identify UTUC protein‐based subtypes via a set of markers able to well characterize the luminal/basal differentiation of the urothelium in both the upper and lower urinary tract. These protein markers have been used in previous studies to stratify UTUC and UBC patients [21, 22, 23, 24] and have shown to correlate well with RNA expression [18, 19]. In addition, we propose a deep‐learning (DL) approach that can predict the identified protein‐based subtypes relying only on digitized hematoxylin and eosin (H&E) slides.

## Materials and methods

### Patient cohorts

The ‘German cohort’ served as training cohort. It comprised formalin‐fixed paraffin‐embedded (FFPE) material of *N* = 163 retrospectively analyzed patients diagnosed with UTUC between 1995 and 2012 at University Hospital Erlangen‐Nürnberg (Erlangen, Germany) and University Hospital Gießen and Marburg (Marburg, Germany). The ‘Dutch cohort’ served as independent test cohort. It comprised *N* = 55 samples diagnosed with UTUC between 2017 and 2020 as part of a multicenter, phase II, prospective trial conducted at University Medical Center Rotterdam (Rotterdam, The Netherlands) [25]. All patients underwent radical nephroureterectomy or partial ureterectomy, without any treatment before surgical tissue collection. All samples were invasive (i.e. with tumor stage ≥ pT1) and for both cohorts one WSI per patient was used, namely the one showing the most representative invasive part of the tumor. All cases were systematically reviewed by two uropathologists (VB and AH) according to the tumor, node, metastasis (TNM) classification (2017) and the WHO classification of genitourinary tumors [26]. Clinicopathological characteristics of the two cohorts are summarized in Table 1. The uropathologists also evaluated slides in terms of histological subtype, infiltration type, and tumor type. Approval for this study was obtained from the Ethics Committee of the Friedrich‐Alexander University Erlangen‐Nürnberg (No. 329_16B) and the Erasmus Medical Centre Rotterdam (METC 2017‐227 NL60919.078.17). All patients gave informed consent. The study was carried out in accordance with the Declaration of Helsinki and to the principles of Good Clinical Practice (GCP).

**Table 1:** Clinicopathological variables characterizing the German and the Dutch cohorts

Clinicopathological variable	German cohort	Dutch cohort
Age at diagnosis (years), median (min–max)	73 (47–94)	71 (52–85)
Gender, *n* (%)		
Female	52 (31.9)	21 (38.2)
Male	111 (68.1)	34 (61.8)
Tumor grade (WHO 1973), *n* (%)		
G1	0 (0)	0 (0)
G2	52 (31.9)	12 (21.8)
G3	111 (68.1)	36 (65.5)
Missing	0 (0)	7 (12.7)
Tumor grade (WHO 2004), *n* (%)	–	
High		54 (98.2)
Low		0 (0)
Missing		1 (1.8)
Tumor grade (WHO 2016), *n* (%)		–
High	142 (87.1)	
Low	21 (12.9)	
Primary tumor, *n* (%)		
pT1	33 (20.2)	17 (30.9)
pT2	28 (17.2)	13 (23.6)
pT3	81 (49.7)	25 (45.5)
pT4	21 (12.9)	0 (0)
Regional lymph nodes, *n* (%)		
pN0	54 (33.1)	22 (40)
pN1	16 (9.8)	3 (5.5)
pN2	14 (8.6)	2 (3.6)
Missing	79 (48.5)	28 (50.9)
Distant metastasis, *n* (%)		
cM0	79 (48.5)	54 (98.2)
cM1	8 (4.9)	0 (0)
Missing	76 (46.6)	1 (1.8)
Anatomic origin, *n* (%)		
Renal pelvis	98 (60.1)	31 (56.4)
Ureter	47 (28.8)	24 (43.6)
Both	18 (11.1)	0 (0)

Estimates are given as median (minimum, maximum) or frequency (percentage) with respect to the total number of analyzed samples (*N* = 163 for the German cohort and *N* = 55 for the Dutch cohort). A dash (−) is used to indicate information not available within a given cohort.

### Tissue microarray analysis

H&E staining and tissue microarray (TMA) analysis were performed for the two cohorts in the respective pathology centers. For each patient a total of four representative tissue cores (1 mm of diameter), two covering the tumor centrum and two covering the invasion front, were punched from the associated paraffin block and transferred to distinct recipient blocks using the TMA Grand Master (3DHistech, Budapest, Hungary).

### IHC analysis

All IHC analyses were performed at the Institute of Pathology, University Hospital Erlangen (Erlangen, Germany) on a Ventana BenchMark Ultra (Ventana, Tucson, AZ, USA) autostainer accredited by the German Accreditation Office (DAKKs) according to DIN EN ISO/IEC 17020.

For protein‐based subtyping, we relied on a set of three basal (i.e. CK14, CK5, and CD44) and three luminal (i.e. CK20, FOXA1, and GATA3) markers that characterize the luminal/basal differentiation of the urothelium in both the upper and lower urinary tract. More specifically, our marker choice was based on the following considerations:

1. the urothelium of both the upper and lower urinary tract can be stratified into three major epithelial cell layers based on their localization and cell type [27]:
  - the basal layer sits on the basement membrane. It is a proliferative cell layer containing stem cells and expressing the basal cytokeratins (CK) 5/6 and CK14, as well as the hyaluronic acid receptor (CD44);
  - the intermediate layer contains moderately differentiated cells with variable expression of CD44, reduced expression of CK5/6, and high expression of CK18;
  - the superficial layer contains the so‐called umbrella cells, which are fully differentiated and express uroplakin proteins as well as CK20.
2. Starting from the differentiation of normal urothelium, urothelial neoplasms develop via two distinct oncogenic pathways [27, 28]:
  - the luminal pathway is driven by the main transcription factors (TFs) GATA3, FOXA1, and PPARG. Cancer cells express markers characteristic of the superficial cell layer;
  - the basal pathway is driven by the TFs p63, STAT2, and EGFR. Resulting cancer cells express markers characteristic of the basal layer.

A subset of the protein markers we utilized have also been used by other groups to stratify UTUC and UBC patients [21, 22, 23, 24]. In addition, the six‐marker set (i.e. CK5, CK14, CD44, FOXA1, GATA3, and CK20) has been extensively used and validated by our group, who showed that the IHC expression of the chosen markers correlates well with RNA expression [18, 19].

IHC staining was performed on 2–3 μm TMA sections from each block using the following antibodies: CK14 (clone SP53, Cell Marque, Rocklin, CA, USA), CK5 (clone XM26, Diagnostic BioSystems, Pleasanton, CA, USA), CD44 (clone DF1485, Dako, Santa Clara, CA, USA), CK20 (clone Ks 20.8, Dako), FOXA1 (polyclonal, Abcam, Waltham, MA, USA), and GATA3 (clone L50‐823, DCS Innovative Diagnostic Systems, Hamburg, Germany). The expression of these markers was histologically quantified (VB and PV) using the histoscore (*H*‐score), which converts immunoreactivity into a semiquantitative range [0–300] proportional to both staining intensity and percentage of positively stained cells [29]. To validate model predictions, immunohistochemical evaluations at whole slide level were performed using the same markers for selected cases.

PD‐L1 expression on immune and tumor cells was assessed on TMAs using the PD‐L1 assay (clone SP263, Ventana) as previously described [30]. Quantification was performed by a pathologist (VB) using both immune cells (IC) score and combined positive score (CPS). The IC score was calculated as the percentage of the area occupied by PD‐L1‐positive IC relative to the total tumor area, whereas the CPS was calculated as the number of immune and tumor cells positive for PD‐L1 out of the total number of tumor cells. Only samples with IC score ≥5% or CPS ≥10 were considered positive for PD‐L1 [31, 32].

### DNA isolation and *FGFR3* SNaPshot analysis

Tumor DNA was isolated using the Maxwell 16 LEV Blood DNA Kit (Promega, Mannheim, Germany) according to the manufacturer's instructions as previously described [33]. *FGFR3* mutational analysis was performed using the SNaPshot method, which simultaneously detects nine hot‐spot mutations, as previously described [34].

### Clustering‐based protein‐based subtype identification and statistical analyses

Hierarchical clustering and statistical analyses were performed within the R environment v.4.0.3 [35]. For protein‐based subtyping, the expression of each luminal/basal marker in each patient was taken equal to the median *H*‐score across the four TMA cores. Unsupervised hierarchical clustering was then performed on the standardized marker expression.

Association between categorical variables was assessed using Fisher's exact test. To compare the distribution of continuous variables, the Wilcoxon rank‐sum test for independent samples (two groups), the Kruskal–Wallis test (more than two groups), or the one‐tailed Wilcoxon signed‐rank test (paired samples) were used. Analyses of overall survival (OS) and disease‐specific survival (DSS) were performed using the Kaplan–Meier estimator, and statistical differences were assessed through the log‐rank test. *p* values <0.05 were considered statistically significant. Further details are provided in Supplementary materials and methods.

### WSI annotation and preprocessing

Slides belonging to the two cohorts were digitized in the respective pathology centers using a Panoramic P250 scanner (3DHistech) at different resolution levels. For each WSI, tumor tissue was manually annotated in QuPath [36] (v.0.2.3) by a trained observer (MA) under expert supervision (VB) (see supplementary material, Figure S1). An automated Python‐based pipeline (https://github.com/MiriamAng/TilGenPro) was implemented to tessellate the identified tumor areas into nonoverlapping tiles of 512 × 512 pixel edge length, perform quality filtering, and stain‐normalization (see supplementary material, Figure S2). Further details are provided in Supplementary materials and methods.

### DL algorithm and its validation

Our DL framework relied on a transfer‐learning approach by fine‐tuning a ResNet50 [37] initialized with weights pre‐trained on the ImageNet database [38]. A repeated three‐fold cross‐validation was used to estimate the model's generalization accuracy and error. Here, to ensure independence between training and validation folds, random splitting was performed at patient level (see supplementary material, Figure S3). To account for class imbalance, the number of tiles belonging to each class within the training set was equalized. The DL model's prediction for single tiles of the validation set was averaged to obtain a WSI‐level subtype prediction. For each repetition, the area under the receiver operating characteristic (AUROC) curve, accuracy, precision, recall, and F1‐score were assessed as mean and 95% confidence interval (CI) across the three validation folds relying on Student's *t*‐distribution. Confusion matrices for a given repetition were obtained by concatenating the predictions for the three validation folds. The predicted class in the independent test cohort was taken equal to the class with the highest average prediction value across the three models from the best‐performing repetition. Further details are provided in Supplementary materials and methods.

## Results

### Hierarchical clustering of protein marker expression identifies luminal, basal, and indifferent UTUC subtypes

**Figure 1:**
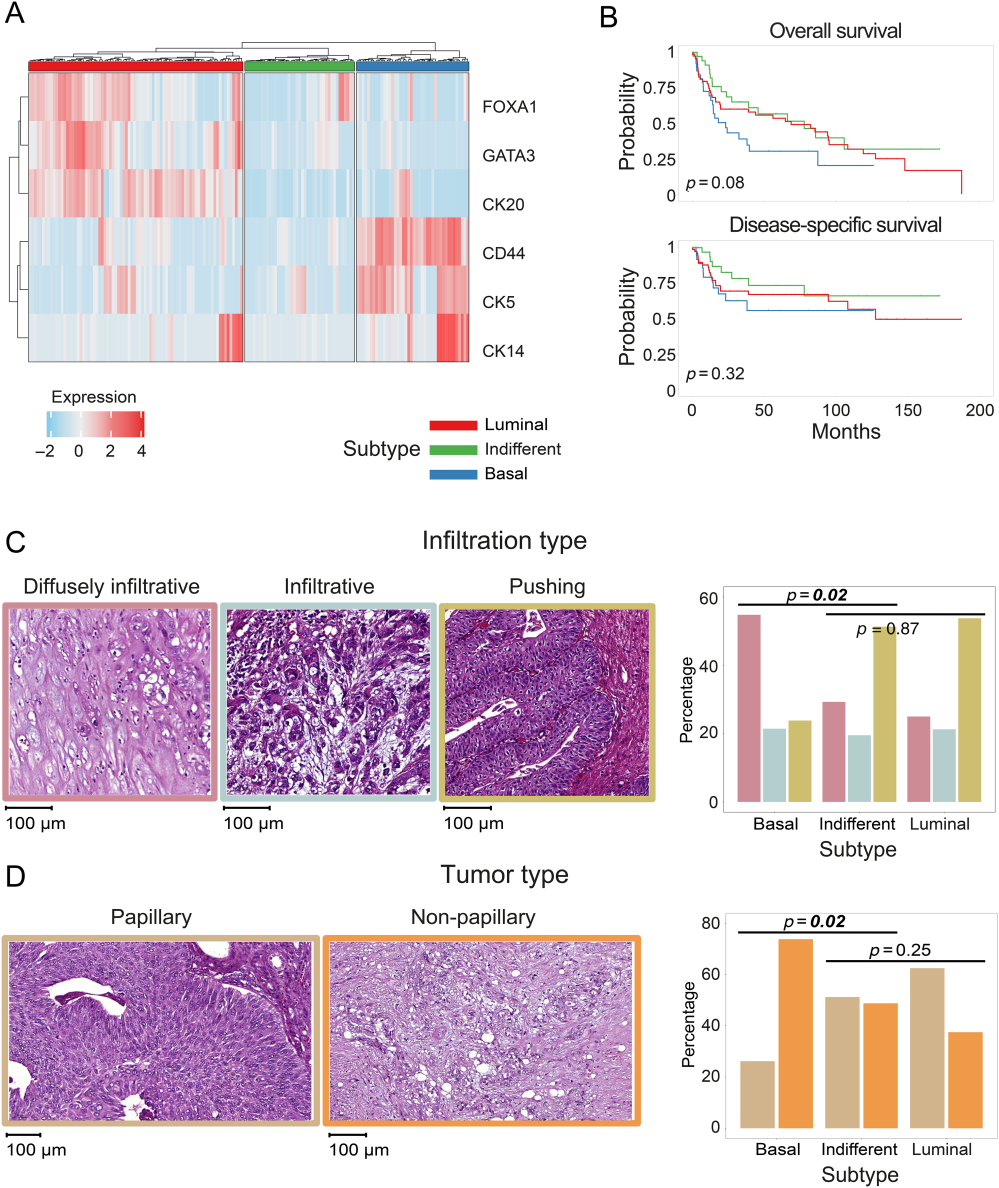
Protein‐based UTUC subtypes and their histopathological characterization. (A) Heatmap visualization of the hierarchical clustering analysis performed on the expression of the three basal (CD44, CK5, and CK14) and three luminal (FOXA1, GATA3, and CK20) markers in our UTUC German cohort (*N* = 163 invasive samples). Heatmap colors represent marker expression (quantified via the standardized *H*‐score, i.e. in terms of standard deviation differences with respect to the average *H*‐score of the marker across all samples; white: equal to the average expression; red: higher than the average expression; blue: lower than the average expression). The color ribbon at the top of the heatmap indicates the three protein‐based subtypes: luminal (red), indifferent (green), basal (blue). (B) Kaplan–Meier overall survival (top) and disease‐specific survival (bottom) curves for the identified subtypes. *p* values from log‐rank test. (C and D) Characterization of the identified subtypes in terms of infiltration type (C) and tumor type (D). Representative histopathology images are shown on the left and bar plot distributions on the right. The *p* values shown above the bar plots refer to two different Fisher's exact tests: one test to compare the distribution of histopathology features in basal versus indifferent subtypes (left) and one test to compare the distribution of histopathology features in luminal versus indifferent subtypes (right). The histopathology images are framed in the respective color used in the bar plot to the right (*p* < 0.05 in italic bold).

To characterize protein‐based UTUC subtypes, the expression of three basal (CK5, CK14, and CD44) and three luminal (CK20, FOXA1, and GATA3) differentiation markers of the urothelium was assessed in a cohort of 163 invasive samples, referred to as the ‘German cohort’. Hierarchical clustering of protein marker expression identified a ‘luminal’ cluster (80 samples, 49.1%), a ‘basal’ cluster (42 samples, 25.8%), and an ‘indifferent’ cluster (41 samples, 25.1%) with low expression of both basal and luminal markers (Figure 1A). Only 2 of the 41 indifferent samples had marker expression equal to zero, while for the others weak expression could be detected.

The basal samples exhibited shorter OS and DSS than the luminal and indifferent samples (Figure 1B). Tumor stages differed across the three subtypes (*p* = 0.01), with almost half of pT4 samples in the basal group (see supplementary material, Table S1). Both infiltration (*p* = 0.02) and tumor type (*p* = 0.02) differed between basal and indifferent cases (Figure 1C,D). In contrast, no significant differences were found when comparing luminal and indifferent subtypes. Indeed, basal samples showed a clear prevalence of diffusely infiltrative and non‐papillary tumors, whereas the other two subtypes showed a higher proportion of pushing and papillary tumors.

Collectively, hierarchical clustering based on the IHC expression of six differentiation markers of the urothelium identified three distinctive protein‐based UTUC subtypes, with high histopathological similarity between the indifferent and the luminal subtype.

### DL successfully predicts luminal and basal protein‐based subtypes from H&E slides and identifies candidate heterogeneous slides

We hypothesized that a DL model could predict the identified protein‐based subtypes using only the information contained in the digitized H&E slides. With this aim, slides were annotated in QuPath (see supplementary material, Figure S1) and preprocessed via an automated Python‐based pipeline (https://github.com/MiriamAng/TilGenPro; see supplementary material, Figure S2). A DL model was then learned on the 163 German samples in a weakly supervised way. Notably, each tile inherited as true class label the protein‐based subtype (i.e. luminal, basal, and indifferent) assigned to the parent slide by hierarchical clustering of the expression of the chosen markers. A total of 100,178 luminal, 66,770 basal, and 57,874 indifferent tiles were used to train and validate a DL‐based classifier in a three‐time repeated three‐fold cross‐validation setting (see supplementary material, Figure S3). While most basal (AUROC = 0.77; 95% CI: 0.67–0.86; repetition three) and luminal (AUROC = 0.71; 95% CI: 0.44–0.99; repetition two) samples were correctly predicted, more than 55% of samples labeled as indifferent on the basis of protein expression were predicted luminal by our DL model on the basis of digitized H&E slides alone (see supplementary material, Figure S4 and Table S2). This difficulty of the DL model in predicting the indifferent subtype was consistent with the observed histomorphological similarity with the luminal subtype.

**Figure 2:**
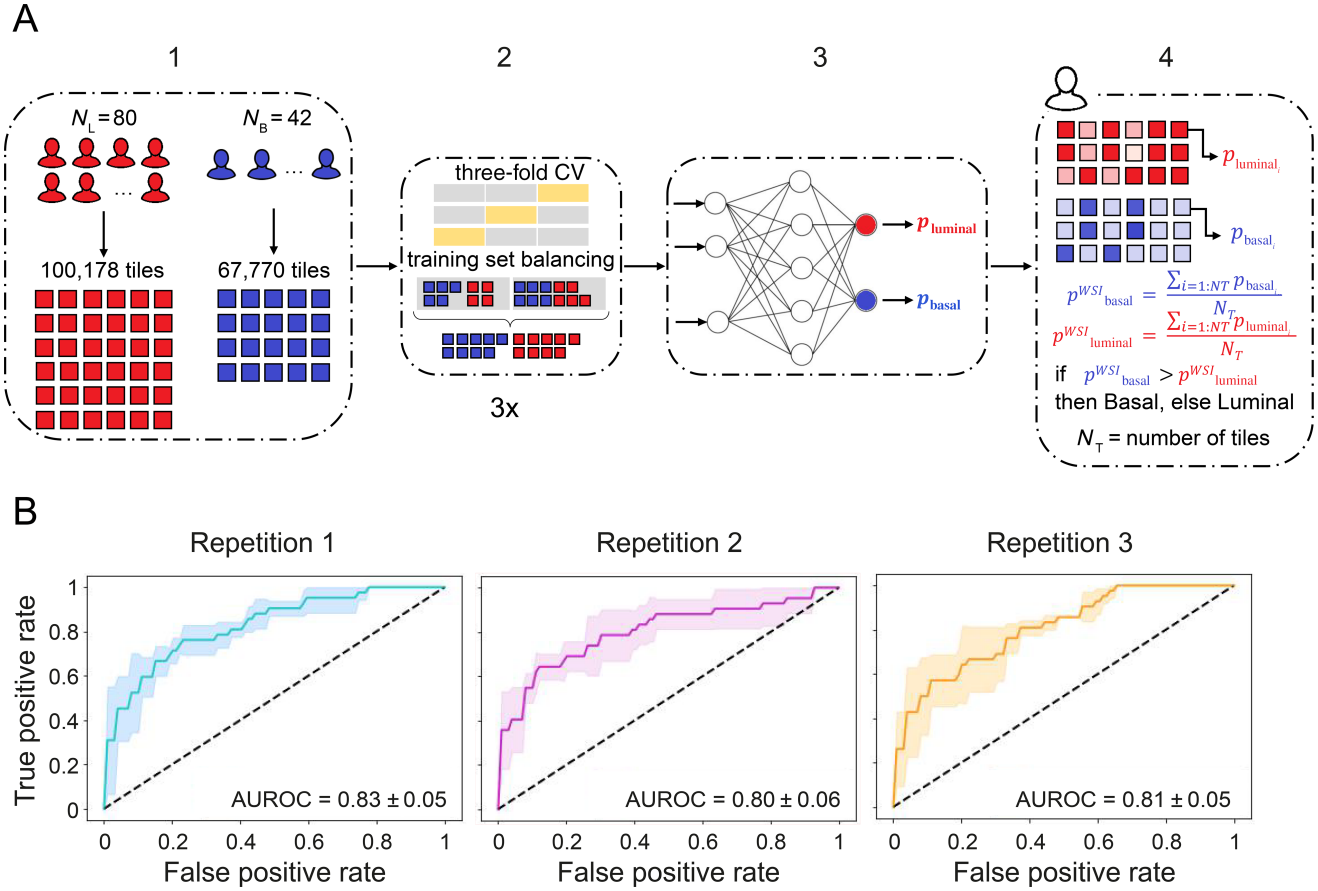
DL‐based prediction of luminal and basal protein‐based subtypes. (A) Steps of the DL framework: (1) Starting from 80 luminal and 42 basal WSIs, a library made up of 100,178 luminal (red) and 66,770 basal (blue) stain‐normalized tiles is generated using an automated, custom‐developed, pre‐processing pipeline. (2) The tiles library is used for training the network using three‐fold cross‐validation (CV) (gray: training folds, yellow: validation fold). Tiles of the trained set are balanced between the two classes. The CV is repeated three times. (3) For each training/validation set combination, a DL model is trained using a transfer‐learning approach. (4) For each tile, the model outputs a prediction value for the luminal (*p*_luminal_) and for the basal (*p*_basal_) class. WSI‐level predictions for the luminal (*p* ^WSI^_luminal_) and basal (*p* ^WSI^_basal_) subtypes are calculated by averaging the tile‐level predictions for each class. The subtype associated with the highest prediction is assigned to the entire slide. In the schematization, color intensity is proportional to the prediction score. (B) AUROC for the three repetitions (with basal subtype as positive class). The mean AUROC ± standard deviation across folds is reported for each repetition. AUROC, area under the receiver operating characteristic; *N*_B_, number of basal slides; *N*_L_, number of luminal slides.

**Figure 3:**
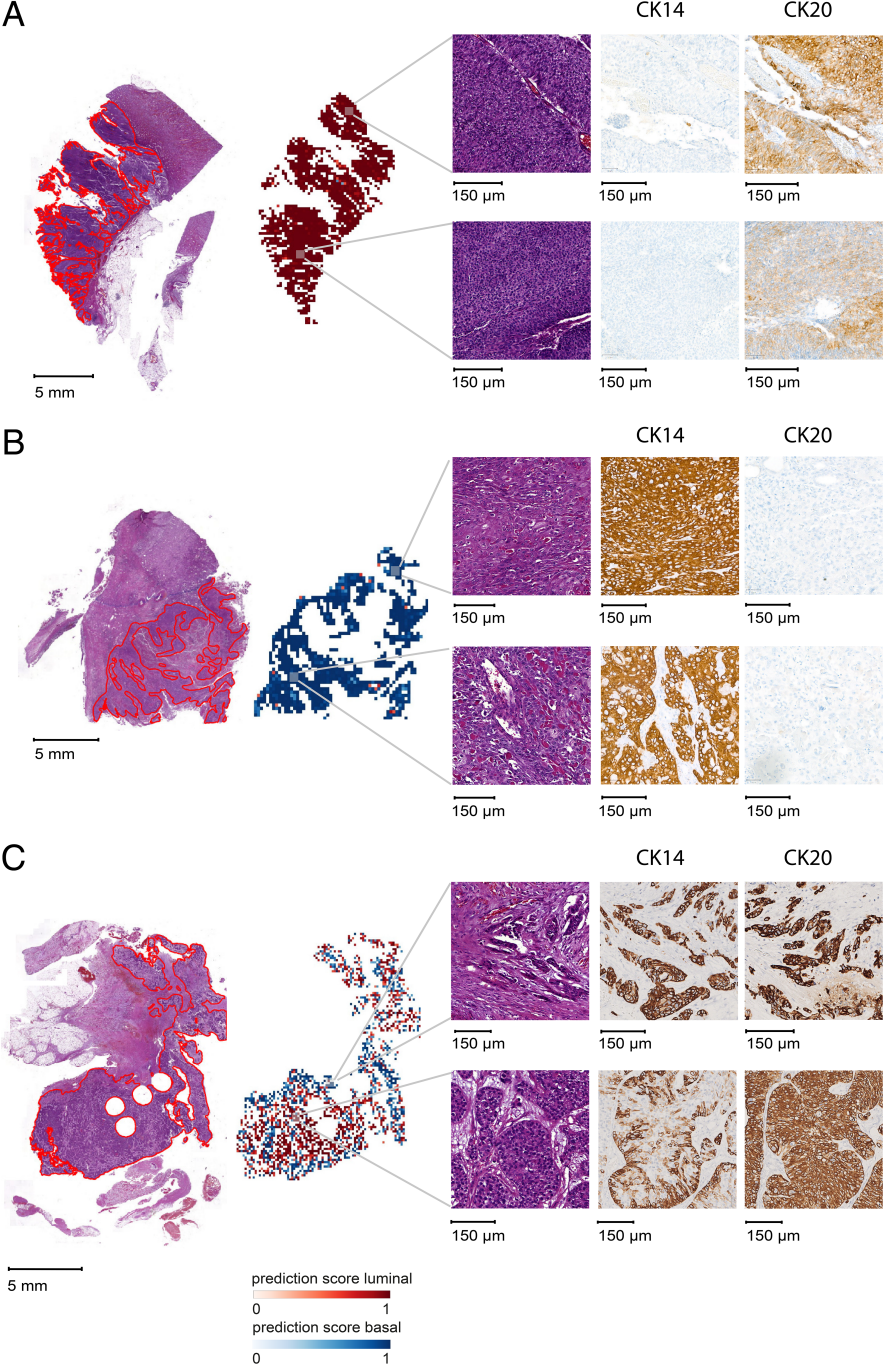
Whole‐slide IHC validation of deep‐learning predictions. (A) Slide predicted with the highest *p* ^WSI^_luminal_, (B) slide predicted with the highest *p* ^WSI^_basal_, and (C) candidate heterogeneous slide. For all three slides, from left to right: digitized whole slide with annotated tumor areas (red); tile‐level prediction map (red: luminal; blue: basal; intensity dependent on prediction score); selected areas; and corresponding areas of the whole‐slide IHC validation using CK20 as representative luminal marker and CK14 as representative basal marker. Whole‐slide IHC validation with all six luminal/basal markers is provided in supplementary material, Figure S5.

Therefore, we decided to train a new DL model focusing on those samples assigned, on the basis of protein expression, to the most distinctive luminal and basal subtypes (Figure 2A). Again, we relied on a repeated cross‐validation setting. Our classifier achieved a mean AUROC of at least 0.8 (AUROC = 0.83; 95% CI: 0.67–0.99; repetition one) and mean accuracy of ≥0.75 (mean accuracy = 0.79; 95% CI: 0.75–0.84; repetition two) (see Figure 2B and supplementary material, Table S3). For further analyses, we focused on the results of repetition 2, as it showed the best accuracy and most consistent performance metrics across the three hold‐out folds (see supplementary material, Table S3). First, the so called ‘high‐confidence’ slides were identified, i.e. those slides whose prediction score for the luminal/basal class, given as output by the model, was at least 0.7 (see supplementary material, Table S4). These slides were selected exclusively on the basis of the DL model prediction scores, irrespective of their protein‐based subtype assigned via clustering of protein marker expression. The true positive rates in the high‐confidence luminal and basal slides were respectively 86.2% (50/58) and 87.5% (14/16). Visual inspection of tile‐level prediction maps of the top high‐confidence slides confirmed the pathological description of these subtypes at histopathological level, i.e. dense nuclei with small stroma bridges as distinctive feature of the luminal subtype (Figure 3A) and dense stroma and keratinization for the basal subtype (Figure 3B) [1]. In the top scoring luminal slide, 99.9% of tiles were predicted luminal. In the top basal, 90% of tiles were predicted basal. These predictions were confirmed by whole slide level staining with the six luminal and basal markers (see Figure 3A,B and supplementary material, Figure S5A,B). Taken together, these results show that the DL model was able to successfully predict the most distinctive luminal/basal protein‐based subtypes on the basis of digitized H&E slides alone.

Next, we focused on the 22 ‘low‐confidence’ slides, i.e. those slides whose prediction score for the luminal/basal class, given as output by the model, was between 0.4 and 0.6 (see supplementary material, Table S5). As for the high‐confidence slides, the low‐confidence slides were chosen exclusively on the basis of the DL model prediction scores, irrespective of their protein‐based subtype. These slides showed no significant difference in the distribution of luminal and basal marker expression (*p* = 0.43). Tile‐level prediction maps allowed categorization of these slides into ‘heterogeneous slides’, with distinguishable clusters of predicted luminal and basal tiles, and slides without any visible luminal/basal structured patterns. Visual inspection of a selected candidate heterogeneous slide supported the predictions, with basal and luminal tiles showing the characteristic histopathological features (Figure 3C). Furthermore, whole slide IHC validation showed positivity of the entire tumor area for the three luminal markers and the basal marker CK14 (see supplementary material, Figure S5C). Notably, in the luminal‐predicted area, the CK14 basal marker appeared expressed only in the outer cellular layer, whereas in the basal‐predicted area it appeared in all cell layers (Figure 3C). Taken together, the DL model was able to identify candidate heterogeneous slides showing co‐presence of luminal and basal areas.

### High‐confidence predicted slides show the expected histopathological features and are significantly associated with PD‐L1 and *FGFR3* status

**Figure 4:**
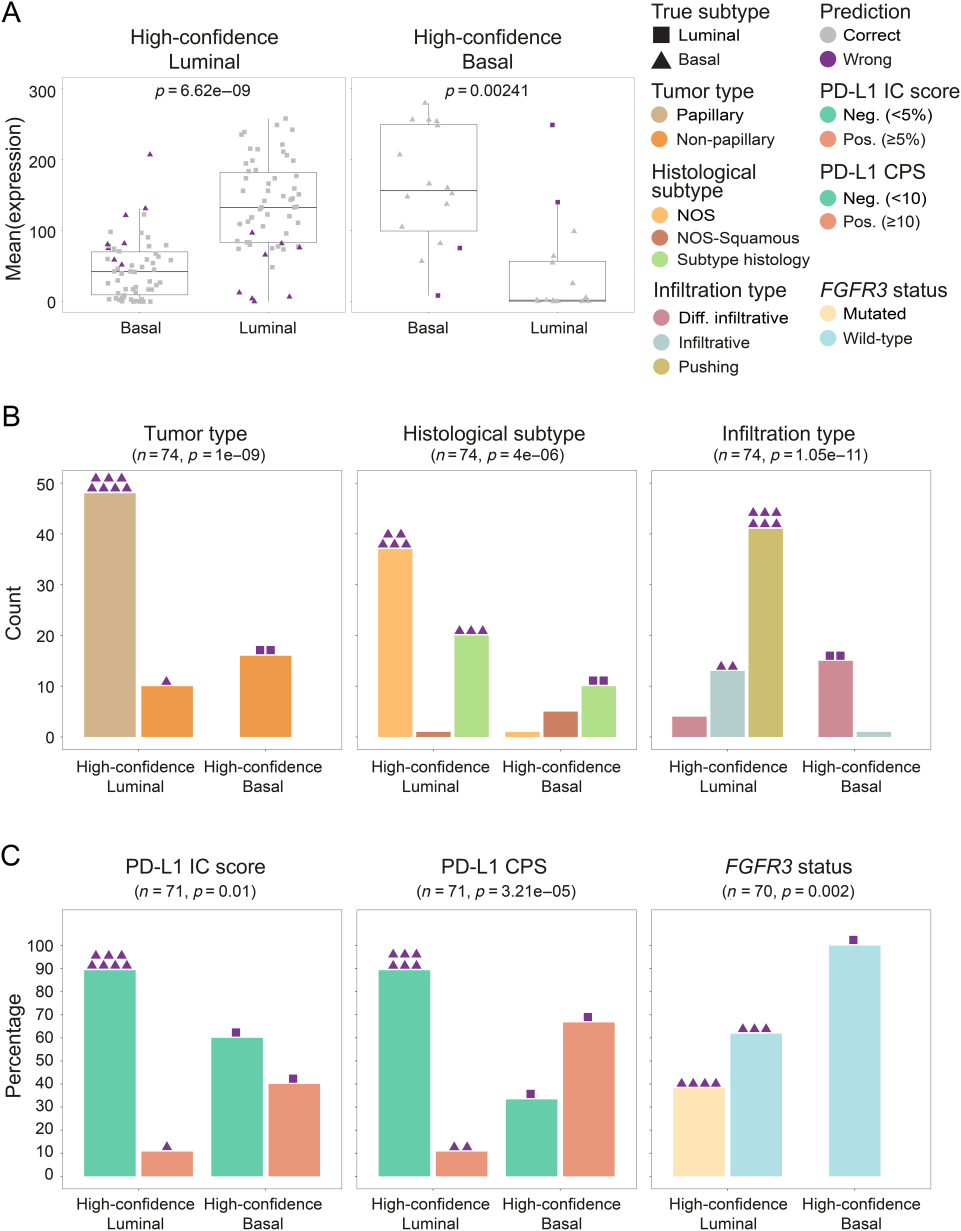
Characterization of the high‐confidence predicted luminal and basal slides. (A) Boxplot distributions of the mean expression values for the basal (CK14, CK5, and CD44) and luminal (CK20, FOXA1, and GATA3) markers in the high‐confidence predicted luminal (left) and basal (right) slides. Shape represents the true slide label (triangle: basal; square: luminal) and color represents the prediction accuracy (gray: correct predictions; violet: incorrect predictions). *p* values from one‐tailed Wilcoxon signed‐rank test. (B) Characterization of the high‐confidence predicted slides in terms of tumor type (left), histological subtypes (middle), and infiltration type (right). An overview of the morphological features for the wrongly predicted slides is provided through the colored symbols above the bars. *n*: number of samples; *p* values from two‐tailed Fisher's exact test. (C) Characterization of the high‐confidence predicted slides in terms of clinically relevant biomarkers: PD‐L1 status (measured as CPS = combined positive score; IC score = immune cells score) and *FGFR3* mutational status. An overview of PD‐L1 and *FGFR3* status of the wrongly predicted slides is provided through the colored symbols above the bars. *n*: number of samples; *p* values from one‐tailed Fisher's exact test.

To further characterize the high‐confidence predictions, we also examined their marker expression and morphological characteristics. High‐confidence predicted luminal and basal slides showed higher expression of luminal (*p* = 6.62 × 10^−9^) or basal markers (*p* = 0.00241) respectively (Figure 4A). High‐confidence luminal predictions were mainly characterized by papillary tumors, with not otherwise specified (NOS) histological subtype, and with pushing infiltration type. Instead, high‐confidence basal predictions were mainly non‐papillary tumors, either squamous or with subtype histology and with diffuse infiltration (Figure 4B). Of note, five of the eight wrongly predicted luminal slides were samples characterized by papillary growth of the tumor with NOS histology. Instead, both wrongly predicted basal slides were diffusely infiltrating, non‐papillary tumors with subtype histology. These results show that high‐confidence predictions showed morphologic features consistent with the DL‐predicted subtype.

Next, we investigated the association of high‐confidence slides with clinically relevant biomarkers (Figure 4C). The proportion of PD‐L1‐positive samples was higher in high‐confidence predicted basal slides, both using IC score (*p* = 0.01) and CPS (*p* < 0.001). Vice versa, the proportion of *FGFR3*‐mutated samples was higher in high‐confidence predicted luminal slides (*p* = 0.002). Interestingly, one wrongly predicted basal slide was actually PD‐L1 positive, whereas four wrongly predicted luminal slides were *FGFR3* mutated.

### External validation of the DL model highlights the importance of subtype histology

To test the generalization ability of the DL model, an external cohort of 55 invasive UTUC patients, referred to as the ‘Dutch cohort’, was used. Hierarchical clustering identified also in this cohort luminal (31 samples, 56.3%), basal (4 samples, 7.3%), and indifferent (20 samples, 36.4%) subtypes, with expression profiles matching those in the German cohort (see supplementary material, Figure S6A). WSI‐level predictions on the Dutch cohort were obtained employing an ensemble of the three DL models trained on repetition 2.

The DL model correctly classified all luminal samples, with an average prediction score of 0.89, but not the four basal samples (see supplementary material, Figure S6B). Three of these samples showed histological subtypes with glandular, squamous, and sarcomatoid features; one sample was instead predominantly characterized by papillary growth of the tumor with NOS histology. However, in this fourth sample, basal features could be observed at the invasion front, where two out of four TMA cores were punched. Interestingly, the tile‐level prediction map highlighted a small tumor area predicted basal in correspondence to the invasion front, whereas the remaining papillary area was mainly predicted luminal (see supplementary material, Figure S6C).

Thus, although a satisfying performance of the DL model was reached in the prediction of the luminal samples, the presence of histological subtypes might have made prediction of the basal samples difficult.

## Discussion

We identified three protein‐based UTUC subtypes via a set of markers that are able to characterize the luminal/basal differentiation of the urothelium in both the upper and lower urinary tract. The three subtypes were identified in a completely unsupervised way, using hierarchical clustering of the protein marker expression. The samples belonging to the indifferent subtype had very low protein expression of both luminal and basal markers, thus clearly differing from both luminal and basal samples. However, our characterization in terms of infiltration type and tumor type highlighted the histopathological similarity between the indifferent and luminal subtypes. This similarity was reflected in the performance of a three‐class DL model, which, utilizing only the digitized H&E slides, predicted as luminal a large number of samples assigned to the indifferent protein‐based subtype. Future studies with molecular data might help to elucidate the molecular‐level differences between the indifferent and the luminal protein‐based subtypes.

Instead, a two‐class DL model trained on only the samples assigned to the luminal and basal protein‐based subtypes could predict with high accuracy these most distinctive luminal/basal subtypes relying only on digitized H&E slides. Furthermore, the DL model identified candidate heterogeneous slides for which whole slide IHC validation confirmed the co‐presence of luminal and basal areas closely matching the tile‐level predictions.

At the histopathology level, invasive UCs present different morphological appearances [1]. As previously observed, MIBC histological subtypes are strong indicators of mRNA‐/protein‐based subtypes [18]. Notably, the high‐confidence predictions by our model, even including those slides where the DL‐predicted subtype did not match the protein‐based subtype, showed morphological features consistent with the DL‐predicted subtype. For example, we saw that five out of the eight WSIs labeled ‘basal’ on the basis of the protein expression, but predicted luminal by our DL model, showed a NOS histology, which is characteristic of the luminal subtype. Moreover, high‐confidence predicted slides were significantly associated with *FGFR3* mutation and PD‐L1 expression. Indeed, basal predictions contained a higher proportion of PD‐L1‐positive samples and luminal predictions a higher proportion of *FGFR3*‐mutated samples. Because of the implementation of anti‐PD‐L1 and PD‐1 therapies, and specific *FGFR3* inhibitors [26, 39] in UCs, histopathology laboratories have been facing increased requests for PD‐L1 assessment and *FGFR3* alteration testing. Our DL model predictions, based exclusively on the information contained in digitized H&E slides, could thus offer valuable support to pathologists for the prioritization of UTUC patients who should undergo *FGFR3*/PD‐L1 testing. This would also contribute to extending patient access to targeted therapies and improve their management and care in clinical practice. Yet, a fully digital diagnostics workflow would be required to implement such prioritization support in daily practice. In addition, testing on even larger UTUC cohorts would be needed.

Several challenges were encountered during our study. Hierarchical clustering in the independent Dutch cohort highlighted the same biological tendency observed in the German cohort, which is even more remarkable considering the prospective nature of the former. This strongly supported the existence of three distinct UTUC protein‐based subtypes. However, the cluster membership of single samples might vary with varying samples being clustered. This uncertainty in the training sample labels might negatively affect the DL model performance. An additional level of uncertainty in training labels was due to the assessment of marker expression in selected TMA cores. It is common practice to stain several tumors at once, while also taking into account tumor heterogeneity. Yet, expression in TMA cores might not be representative of whole slide level expression, as clearly seen for the predominantly papillary case in the Dutch cohort with a basal‐like morphology at the invasion front. RNA‐sequencing analyses might offer more robust subtype assignment, thanks to genome‐wide profiling, yet might still fail to correctly characterize heterogeneous samples. Another challenge was linked to histological subtypes. Given the rarity of UTUC, we had decided not to exclude them, as had been done in previous UBC studies [19]. However, as the results on the Dutch cohort showed, this might have impaired model performance. Histological subtypes have gained increasing importance, given their impact on pathological and clinical outcomes [40]. Thus, it would be very important to develop machine learning approaches able to accommodate the prediction of histological subtypes from H&E slides. This might be achievable with the future availability of an even more extensive UTUC cohort, with a sufficient number of samples for all histological subtypes to ensure robust training of a DL model.

Furthermore, in the future it would be very interesting to investigate the extension of our DL framework to biopsy samples. Here, challenges will be related to whether an intrinsically small and superficial biopsy sample provides enough information to predict subtypes and ultimately offers support in deciding on the best treatment strategy. Finally, additional steps could be integrated into the workflow to facilitate the use of the developed DL model in a fully digital pathology laboratory. First, an upstream tumor segmentation step could be implemented to avoid the manual annotation of new samples. In addition, it would be useful to develop a downstream postprocessing tool for the automatic detection of candidate heterogeneous slides based on luminal/basal patterns analysis of tile‐level prediction maps.

Collectively, our results show that the most distinctive protein‐based UTUC subtypes can be predicted directly from H&E slides and are associated with the presence of targetable alterations. Thus, our study lays the foundation for an AI‐based tool to support UTUC diagnosis and extend patient access to targeted treatments.

## Author contributions statement

MA, FF and VB conceived and designed the study. MA, TvD, PV, JS, RS, CIG, HH, SW, HT, DS, BW, GJLHvL, ME, JLB, FF and VB acquired the data. MA, SL, FF and VB analyzed and interpreted the data. MA and FF performed the statistical analyses. MA, FF and VB drafted the manuscript. ME, AH, JLB and FF obtained funding. SF, CM and VZ provided resources. All authors critically revised the manuscript for important intellectual content, read and approved the final manuscript.

## Supporting information


**Supplementary materials** **and methods**
**Figure S1.** Example of tumor annotation
**Figure S2.** Overview of the WSI pre‐processing workflow
**Figure S3.** Overview of the training set generation procedure in a three‐fold cross‐validation setting
**Figure S4.** Deep‐learning model performance in the prediction of the luminal, basal, and indifferent subtypes
**Figure S5.** Whole slide IHC validation with the entire marker set of the samples shown in Figure 3
**Figure S6.** Validation of the deep‐learning model on the Dutch cohort
**Table S1.** Association analysis of the identified subtypes with the main clinicopathological variables
**Table S2.** Performance metrics of the deep‐learning classifier in the prediction of the luminal, basal, and indifferent protein‐based subtypes for the three repetitions
**Table S3.** Performance metrics of the deep‐learning classifier in the prediction of the luminal and basal protein‐based subtypes for the three repetitions

**Table S4.** High‐confidence predicted luminal and basal slides

**Table S5.** Low‐confidence predicted slides

## Data Availability

The data supporting the findings of this study are available from the corresponding authors upon reasonable request.
