# Supplementary material for: A deep‐learning workflow to predict upper tract urothelial carcinoma protein‐based subtypes from H&E slides supporting the prioritization of patients for molecular testing
Source: J Pathol Clin Res. 2024 Mar 19;10(2):e12369. doi: 10.1002/2056-4538.12369 (PMC10951050; doi:10.1002/2056-4538.12369)
Supplement: Supplementary file 1 — Supplementary materials and methods Figure S1. Example of tumor annotation Figure S2. Overview of the WSI pre‐processing workflow Figure S3. Overview of the training set generation procedure in a three‐fold cross‐validation setting Figure S4. Deep‐learning model performance in the prediction of the luminal, basal, and indifferent subtypes Figure S5. Whole slide IHC validation with the entire marker set of the samples shown in Figure 3 Figure S6. Validation of the deep‐learning model on the Dutch cohort Table S1. Association analysis of the identified subtypes with the main clinicopathological variables Table S2. Performance metrics of the deep‐learning classifier in the prediction of the luminal, basal, and indifferent protein‐based subtypes for the three repetitions Table S3. Performance metrics of the deep‐learning classifier in the prediction of the luminal and basal protein‐based subtypes for the three repetitions [file CJP2-10-e12369-s002.pdf]

# A deep-learning workflow to predict upper tract urothelial carcinoma protein-based subtypes from H&E slides supporting the prioritization of patients for molecular testing

M Angeloni *et al., J Pathol Clin Res*, https://doi.org/10.1002/2056-4538.12369

**Supplementary materials and methods**

**Supplementary Figures S1–S6**

**Supplementary Tables S1–S5** (see separate Excel files for Tables S4 and S5)

Reference numbers refer to the list in the main paper.

## Supplementary Materials and Methods

### Clustering-based protein-based subtypes identification and statistical analyses

Hierarchical clustering and statistical analyses were performed within the R environment v.4.0.3 [35]. To identify protein-based subtypes, the expression of each marker in each patient was taken equal to the median H-score across the four TMA cores. Unsupervised hierarchical clustering was performed on the standardized marker expression (i.e. scaled to a mean of zero and a standard deviation of one) using Ward's clustering method [41]. Ward's algorithm was implemented relying on the R function *hclust* using as input the dissimilarity matrix computed through the Euclidean distance and ward.D2 as argument for the agglomeration method [42]. Heatmap visualization of the hierarchical clustering analysis was performed relying on the function *Heatmap* from the R-package ComplexHeatmap v.2.4.3.

Association analysis between categorical variables was performed using Fisher's exact test. To compare the distribution of continuous variables, the Wilcoxon rank-sum test for independent samples (two groups) or the Kruskal-Wallis test (more than two groups) were used. Differences in the distribution of luminal and basal marker expression were evaluated using the one-tailed Wilcoxon signed-rank test for paired samples.

Analyses of overall survival (OS) and disease-specific survival (DSS) were performed using the Kaplan-Meier estimator relying on the R-packages *survminer* v.0.4.9 and *survival* v.3.2-13. The statistical difference between survival curves was assessed through the log-rank test. P-values (p) $< 0.05$ were considered statistically significant.

### Slides digitization and WSI annotation

Slides belonging to the two cohorts were digitized in the respective pathology centers using a Panoramic P250 scanner (3DHistech, Budapest, Hungary). Glass slides from the German cohort were scanned at 20-fold magnification with a resolution of 0.389 microns per pixel (mpp) whereas those from the Dutch cohort had three different resolution levels, i.e. 0.1214 mpp at 40-fold magnification, 0.2428 mpp at 20-fold magnification and 0.2484 mpp at 40-fold magnification. To facilitate the analysis and pre-processing of WSIs, digitized slides from the two cohorts were organized into two distinct QuPath [36] (v.0.2.3) projects and stored as .qpproj files. Within each project, for each WSI the tumor tissue was manually annotated by a trained observer (MA) under the supervision of an expert uropathologist (VB).

Manual annotation consisted in drawing a region of interest (ROI) around the tumor area and leaving out healthy tissue. Annotations were made at high levels of magnification using the brush and wand tools available in QuPath to exclude as much as possible non-tumor tissue including necrosis, bleeding/blood vessels, peri-tumoral lymphocytes and scanning artifacts (Figure S1).

### WSI pre-processing pipeline

WSIs pre-processing as well as deep-learning (DL) analyses were performed in Python v.3.7.12 and run in a dedicated conda environment on a remote server based on Ubuntu's 20.04.5 long-term support (LTS) operating system with NVIDIA Tesla V100-PCIE-32GB graphics processing unit (GPU).

An automated Python-based pipeline (https://github.com/MiriamAng/TilGenPro) was implemented for the pre-processing of WSIs. The pipeline only requires as input a QuPath project of annotated WSIs. First a

groovy script is run to tessellate the annotated tumor areas into smaller non-overlapping square patches, a.k.a. tiles, of 512x512 pixel edge length. Subsequently, the generated tiles undergo a quality-filtering step. Namely, for a given WSI the median pixel intensity value across the RGB channels is calculated for each of the belonging tiles and a $\log_{10}$-transformed median pixel intensities distribution is obtained. A lower/upper percentile-based threshold can thus be set on the obtained distribution to filter-out tiles with a $\log_{10}$ median intensity lower or equal than the lower threshold (this corresponds to tiles characterized by darker regions) and/or greater or equal than the upper threshold (this corresponds to tiles with a high amount of white pixels). The pipeline was run using as values for the lower/upper thresholds the $5_{th}$ and $90_{th}$ percentiles respectively. Finally, to reduce stain variation between training and test set, the tiles passing the quality-filtering step are stain-normalized according to the Macenko method [43] (Figure S2). Macenko stain-normalization was implemented by assigning to the input parameters α and β the values of 1 and 0.15 respectively, as recommended by the authors [43], and using as reference H&E optical density (OD) matrix the one provided by Mitko Veta's 'Staining unmixing and normalization' code (https://github.com/mitkovetta/staining-normalization). For the Dutch cohort, non-overlapping tiles of 512x512 pixel edge length were generated with the same resolution level as the German cohort (i.e. 0.389 mpp).

A total of 341,906 (mean: 2,098; range: 89-7,514; 146,611 luminal; 109,681 basal; 85,614 indifferent) and 112,562 (mean: 2,047; range: 58-4,964; 57,319 luminal; 10,258 basal; 44,985 indifferent) tiles were generated for the German and the Dutch cohort respectively. To control, in training dataset composition, the over-representation of tiles from WSIs characterized by larger tumor areas, and instead maximize the representativeness of tiles associated with smaller tumor areas, a

maximum number of 2,000 tiles was randomly subsampled from the WSIs of the German cohort during training.

**Deep-learning algorithm and its validation**

For protein-based UTUC subtypes prediction a 'classical weakly supervised' [44] DL framework was employed. Namely, all tiles originating from a given WSI inherited the corresponding patient-level label assigned by hierarchical clustering [44, 45]. Although in real life tumor homogeneity cannot be assumed, weakly supervised learning approaches are particularly suited to the computational pathology field, whenever DL algorithms aim at predicting labels that cannot be directly annotated on digitized H&E slides [19, 46, 47]. Indeed, clinically relevant labels (e.g. mutational status or subtypes) are known only at patient level, while DL model training is performed on smaller image tiles generated from the WSI. The goal of the developed DL model is to make a prediction of the protein-based subtypes identified via hierarchical clustering using only the information contained in the digitized H&E slides. In order to achieve this, the DL model must 'learn'. During model learning, the dataset with known class labels (in our case, the German cohort) is split into two portions: a training portion and a validation portion. The training portion is passed to the model together with the known class labels. The known class labels are necessary to quantify how much a given prediction deviates from its true class and this information is used to make the model learn via parameters tuning. The validation portion is instead used to provide an early estimate on how well the model learned and tune the model's hyper-parameters [48]. Often a cross-validation framework is utilized, where a dataset is divided into n folds (portions) and n DL models are learnt, each time using the i-th fold (i=1 … n) as validation portion, a.k.a.

hold-out fold, and the remaining (n-1) folds as training portion.

To predict protein-based subtypes in UTUC we chose a ResNet50 [37]. The ResNet50, initialized with weights pre-trained for the visual recognition challenge on the ImageNet database [38], was then fine-tuned for the specific task relying on a transfer-learning approach. For model's implementation the DL library *fastai* v.1.0.61 [49], which is built on top of the open source PyTorch machine-learning framework [50], was employed. The pre-trained ResNet50 was retrieved from the *vision.learner* fastai module. This module, through the cnn_learner method, allows to easily retrieve a pre-trained model with a head suitable for the specific classification task.

To adapt the pre-trained model to the classification of protein-based subtypes, only the model's head was fine-tuned while keeping the layers of the backbone frozen. Model fitting was performed relying on the 1-cycle training policy [51] through the fastai function *fit_one_cycle*, by setting a maximum number of 30 epochs, a maximum learning rate of $10^{-5}$, and a weight decay of 0.1. In addition to weight decay, other regularization techniques were implemented to avoid overfitting, including data augmentation and early stopping. Data augmentation was performed relying on the fastai *get_transforms* function using the default random transformations (i.e. horizontal flipping, rotation, zooming, warping, and lighting). To introduce rotational invariance also vertical flipping was adopted by setting to true the argument flip_vert. For early stopping implementation validation accuracy was chosen as quantity to be monitored throughout the whole training process. Notably, the fastai early stopping callback was implemented to terminate training after a patience time of three epochs with no improvement (min_delta = 0.01) of the monitored metric. The fastai save model callback was then used to save the model at the best epoch, i.e. the best model.

A three-time repeated three-fold cross-validation was used to estimate the model's generalization accuracy and error. Here, to ensure independence between training and validation sets, the random splitting into the three folds was performed at patient level. Further, the splitting was performed in a stratified manner, i.e. preserving the percentage of samples for a given class within each partition (Figure S3). To this aim, we relied on the module *StratifiedKFold* from the scikit-learn package v.0.24.1 using a different value of the random state argument for each repetition and setting to true the shuffle parameter. At each round of the cross-validation, the DL model was trained on two folds out of three and evaluated on the hold-out fold. Run times to fully train the DL model in a three-time repeated three-fold cross-validation setting were around 1.5 day. To account for class imbalance, a tiles balancing procedure was implemented to equalize the number of tiles belonging to each class within the training set. During inference, a prediction value per subtype was assigned to each image tile. For each WSI, tile-level predictions were then averaged, class-wise, and the subtype predicted with the highest average prediction was assigned to the WSI.

Performance metrics to evaluate model's performance were calculated relying on the sklearn.metrics module. The area under the receiver operating characteristic (AUROC), accuracy, precision, recall, and F1-Score were assessed for each repetition as mean across the three hold-out folds and 95% confidence interval (CI) relying on Student's t-distribution. Confusion matrices for a given repetition were instead obtained using the concatenated model's predictions on the associated hold-out folds.

As final model for the independent test cohort, a cross-validation ensemble was used. Namely, for each WSI of the Dutch cohort WSI-level predictions were

obtained using each of the three models from the best-performing repetition on the German cohort. Then, the final WSI-prediction was taken as the class (luminal/basal) with the highest average prediction value across the three models.

**Tiles balancing procedure**

For a classification problem involving $N$ different classes ($c_i$, with $i = 1, \ldots, N$), our proposed tiles balancing procedure relies on the following steps:

1. calculate the number of tiles to be balanced $t^*$ as the sum of the number of tiles belonging to the class with the lower number of associated WSIs (in case of two or more classes with the same number of associated WSIs choose the class with the lower number of associated tiles);
2. given $Nw_{c_i}$ the total number of WSIs belonging to each of the remaining $N-1$ classes, calculate the maximum number of tiles ${t^*}_{c_i}$ to

   keep for each WSI belonging to class $i$ as ${t^*}_{c_i} = {t^*}/{Nw_{c_i}}$. If all the $Nw_{c_i}$ WSIs have a number of tiles > ${t^*}_{c_i}$, random subsample ${t^*}_{c_i}$ tiles. The balancing procedure is over.
3. If not all the $Nw_{c_i}$ WSIs have a number of tiles > ${t^*}_{c_i}$, let $Nw_{c_i}^{lowEq}$ be the number of WSIs of class $i$ with a number of tiles $\leq {t^*}_{c_i}$ and $Nw_{c_i}^{high}$ the number of WSIs of class $i$ with a number of tiles $> {t^*}_{c_i}$.

   Given ${t^*}_{c_i}(j)$ the number of tiles belonging to the $j$-th WSI of class $i$:

   a. keep all the tiles associated with the $Nw_{c_i}^{lowEq}$ WSIs and calculate the first contribution to the balancing as $t_{c_i}^{lowEq} = \sum_{j=1}^{Nw_{c_i}^{lowEq}} t_{c_i}(j)$;
   b. calculate the new number of tiles ${t^{high*}}_{c_i}$ to keep for the $Nw_{c_i}^{high}$ WSIs

as $t^{high*}{}_{c_i} = \frac{t* - t_{c_i}^{lowEq}}{Nw_{c_i}^{high}}$ . If a WSI has a number of tiles $<=$ $t^{high*}{}_{c_i}$ keep them all (second contribution to the balancing for class $i$: $t_{c_i}^{high1}$), otherwise random subsample $t^{high*}{}_{c_i}$ tiles (third contribution to the balancing for class $i$: $t_{c_i}^{high2}$).

The balancing procedure will satisfy the following equation:

$$t^* \cong t_{c_i}^{lowEq} + t_{c_i}^{high1} + t_{c_i}^{high2}, for\ i = 1, \ldots, N-1$$

## Supplementary Figures

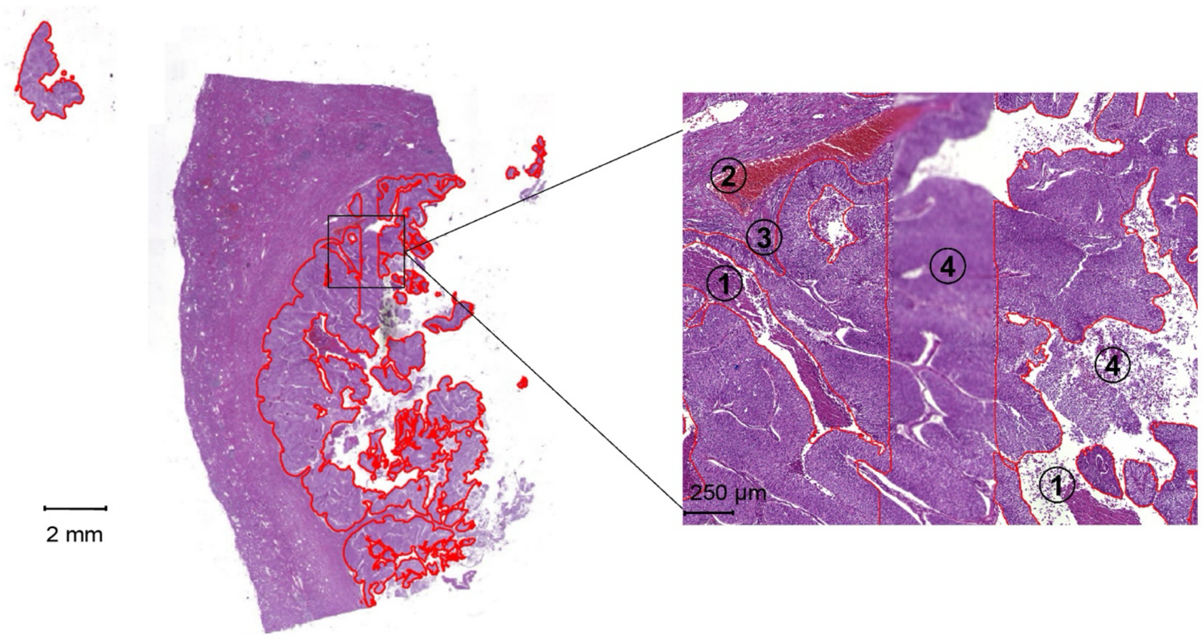


**Figure S1. Example of tumor annotation.** (left) Overview of a digitized whole slide image (WSI) with tumor tissue annotation borders in red; (right) zoomed-in tumor area representative of the employed annotation criteria: necrosis (1), bleeding/blood vessels (2), peri-tumoral lymphocytes (3) and scanning artifacts such as blurring and poor fixation (4) were excluded from the annotated area.

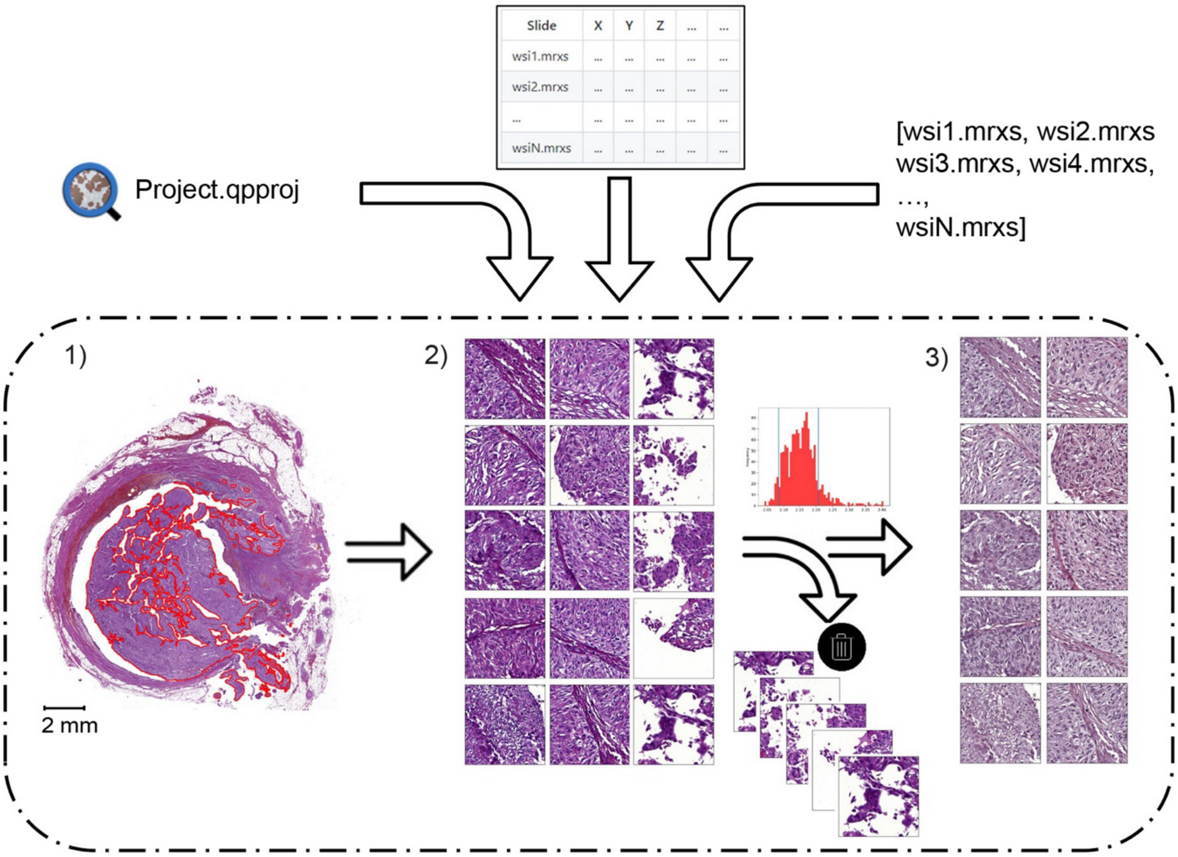


**Figure S2. Overview of the WSI pre-processing workflow.** Graphical representation of the main steps performed by the automated Python-based pre-processing pipeline. A QuPath project (Project.qpproj) or a set of WSIs to process (provided via a csv file, or specified individually as a list of one or more WSI names) can be used as input for the pipeline. The pipeline performs the following steps: (1) the annotated tumor area (red) within each slide is tessellated into smaller tiles; (2) tiles undergo a quality-filtering step based on lower/upper thresholds identified on the log10-transformed median intensity values distribution; (3) tiles are stain-normalized according to the Macenko method.

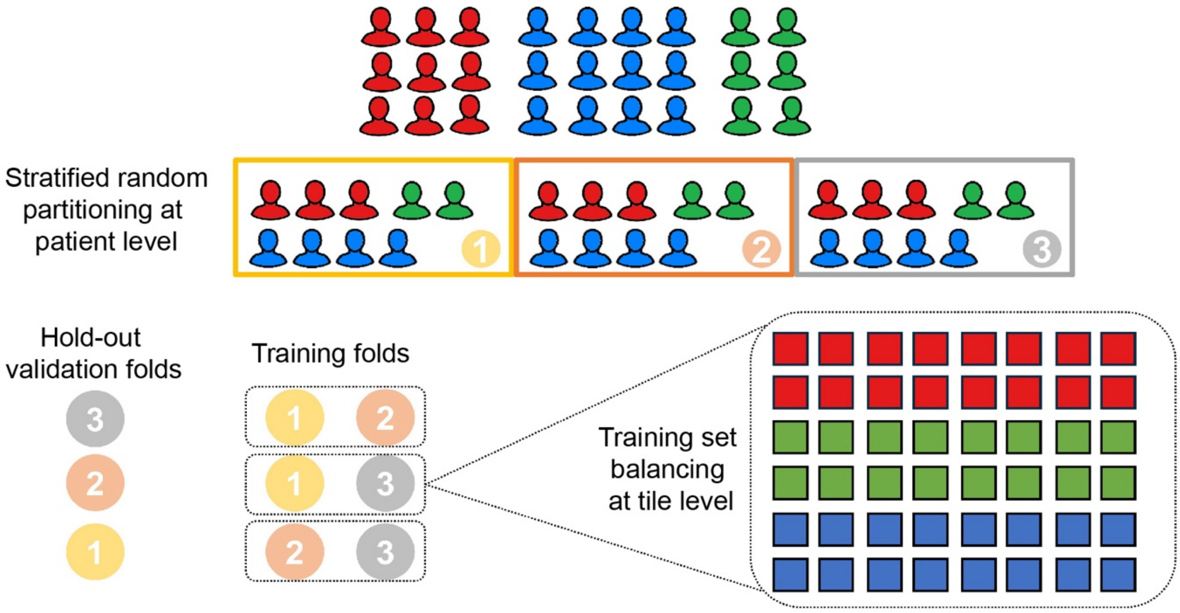


**Figure S3. Overview of the training set generation procedure in a three-fold cross-validation setting.** In the example, each icon represents a patient (in our case corresponding to a slide, as only one slide per patient was used) of the analyzed cohort and the three colors (red, blue and green) represent three different classes of patients. The patients' cohort is randomly split in three stratified partitions, i.e. with the same class distribution as in the whole cohort. Two partitions out of three are used, in turn, to train the deep-learning model while the correspondent hold-out folds are used for validation. Tiles belonging to the two training partitions are pooled together and class-balanced. In the example tiles are represented by the square symbol and inherit the same color (i.e. class) of the parent patient.

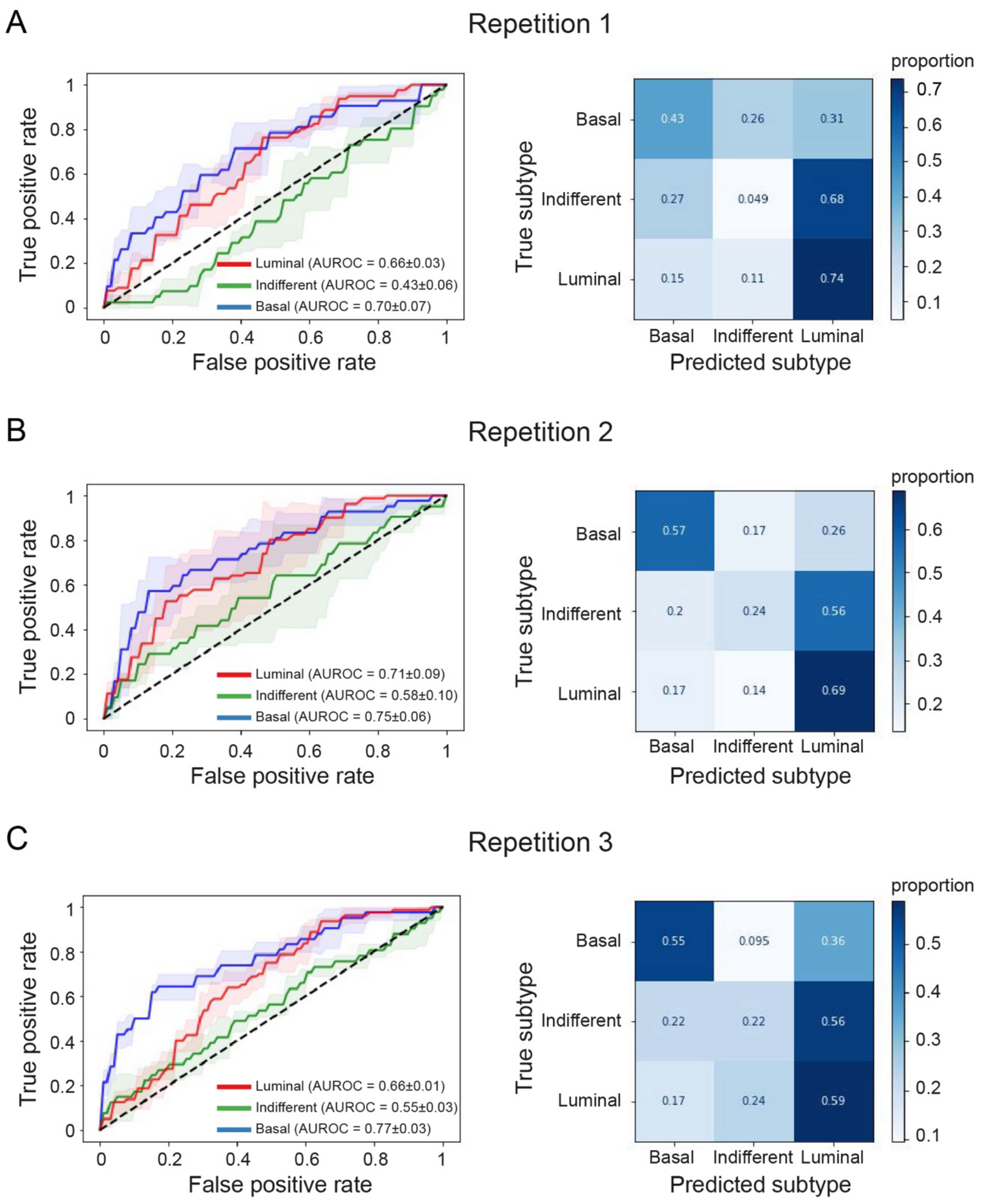


**Figure S4. Deep-learning model performance in the prediction of the luminal, basal, and indifferent subtypes.** (left) AUROC curves and (right) confusion matrices for (A) repetition 1, (B) repetition 2 and (C) repetition 3 of the three-fold cross-validation. AUROC curves are shown for each subtype (blue: basal, green: indifferent, red: luminal). The mean AUROC ± standard deviation (sd) is reported for each repetition. Confusion matrices are normalized over the true class (row). AUROC: area under the receiver operating characteristic.

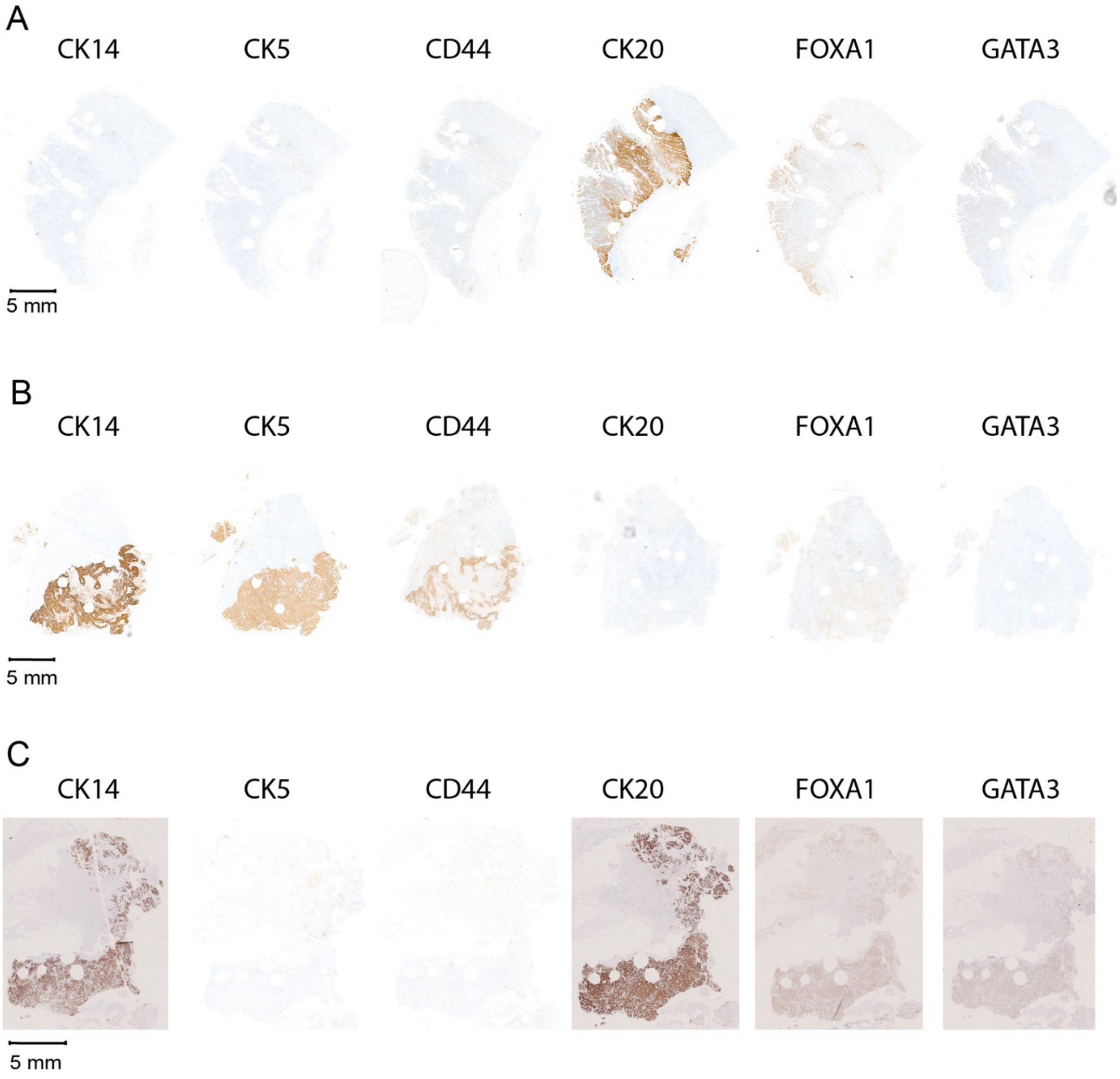


**Figure S5. Whole-slide IHC validation with the entire marker set of the samples shown in Figure 3.** Whole-slide IHC-validation with the three basal (CK14, CK5, CD44) and three luminal (CK20, FOXA1, GATA3) markers for (A) the top high-confidence predicted luminal slide, (B) the top high-confidence predicted basal slide, and (C) a candidate heterogeneous slide.

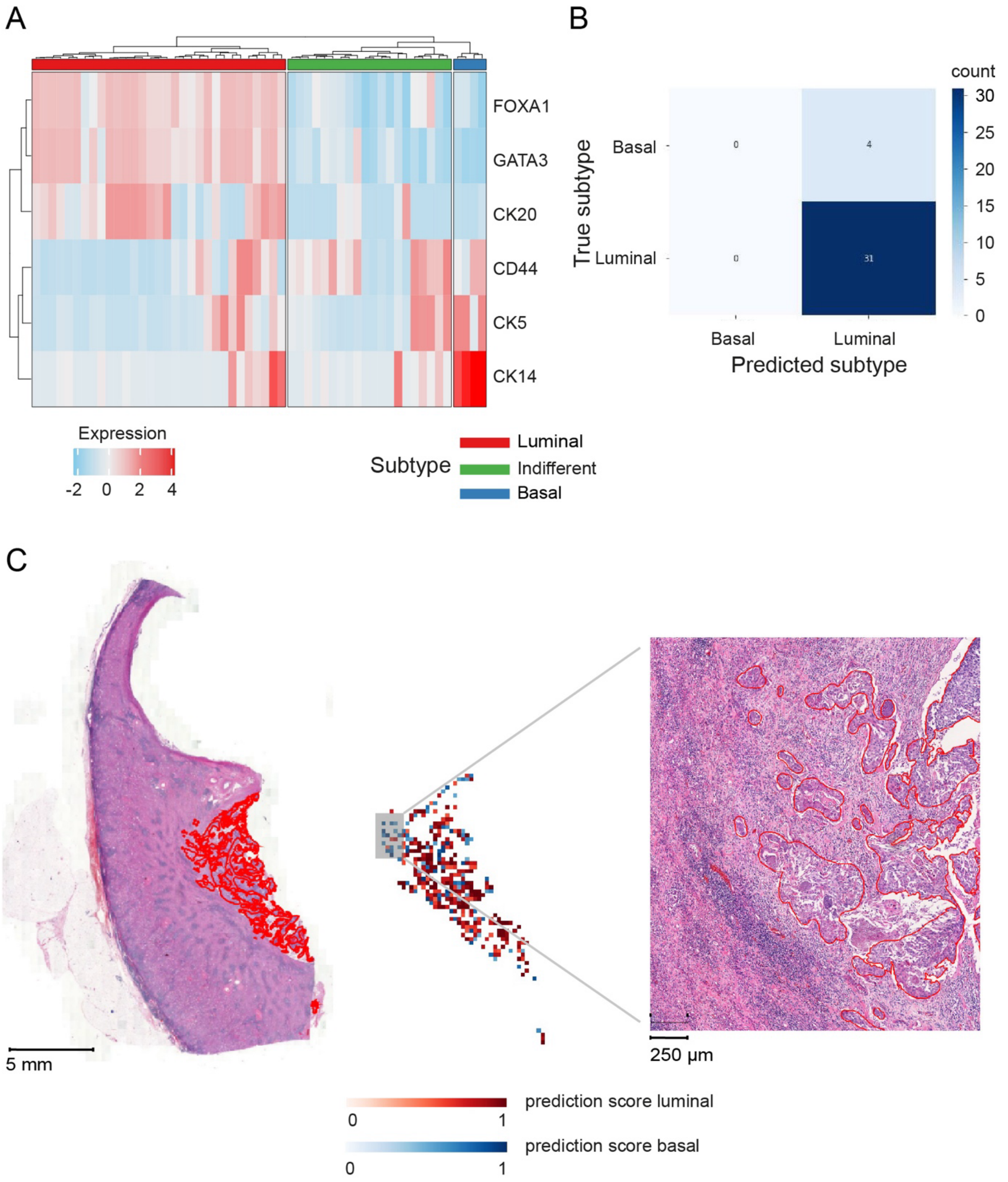


**Figure S6. Validation of the deep-learning model on the Dutch cohort.** (A) Heatmap visualization of the hierarchical clustering analysis performed on the expression of the three basal (CD44, CK5, CK14) and three luminal (FOXA1, GATA3, CK20) markers in the independent UTUC cohort from the Netherlands (N = 55 invasive samples). Heatmap colors represent marker expression (quantified via the standardized H-score, i.e. in terms of standard deviation differences with respect

to the average H-score of the marker across all samples; white: equal to the average expression; red: higher than the average expression, blue: lower than the average expression). The color ribbon at the top of the heatmap indicates the three protein-based subtypes: luminal (red), indifferent (green), basal (blue). (B) Confusion matrix with a summary of model's generalization accuracy. (C) Selected basal slide predicted luminal by the model. From left to right: digitized whole-slide image (WSI) with annotated tumor areas (red); tile-level prediction map (red: luminal, blue: basal; intensity dependent on prediction score); zoomed area for a better visualization of the basal morphological features of the invasion front.

## Supplementary Tables

**Table S1. Association analysis of the identified subtypes with the main clinicopathological variables.** Estimates are given as median (minimum, maximum) or frequency (percentage) calculated on the total available samples. Kruskal-Wallis and Fisher's exact tests were used respectively for continuous and categorical variables.

| Clinico-pathological variable | Luminal | Indifferent | Basal | p-value |
|---|---|---|---|---|
| *Age at diagnosis (yrs),* median (min-max) | 72 (47-94) | 70 (48-85) | 77 (51-87) | ***0.02*** |
| *Gender,* n (%) | | | | 0.07 |
| F | 24 (30) | 9 (22) | 19 (45.2) | |
| M | 56 (70) | 32 (78) | 23 (54.8) | |
| *Tumor Grade (WHO 2016),* n (%) | | | | 0.18 |
| low | 12 (15) | 7 (17.1) | 2 (4.8) | |
| high | 68 (85) | 34 (82.9) | 40 (95.2) | |
| *Primary Tumor,* n (%) | | | | ***0.01*** |
| pT1 | 20 (25) | 10 (24.4) | 3 (7.1) | |
| pT2 | 19 (23.8) | 4 (9.8) | 5 (11.9) | |
| pT3 | 36 (45) | 21 (51.2) | 24 (57.2) | |
| pT4 | 5 (6.2) | 6 (14.6) | 10 (23.8) | |
| *Regional Lymph Nodes,* n (%) | | | | 0.11 |
| N0 | 31 (77.5) | 11 (47.8) | 12 (57.1) | |
| N1 | 4 (10) | 6 (26.1) | 6 (28.6) | |
| N2 | 5 (12.5) | 6 (26.1) | 3 (14.3) | |
| *Distant Metastasis,* n (%) | | | | 0.57 |
| M0 | 36 (87.8) | 23 (95.8) | 20 (90.9) | |
| M1 | 5 (12.2) | 1 (4.2) | 2 (9.1) | |

F: female; M: male. Statistically significant p-values (<0.05) are highlighted in italic bold.

**Table S2. Performance metrics of the deep-learning classifier in the prediction of the luminal, basal, and indifferent protein-based subtypes for the three repetitions.** For each repetition a measure of precision, recall, F1-Score, and AUROC is reported separately for the luminal, basal, and indifferent subtype together with the overall accuracy across the three subtypes. All performance metrics are reported as mean [95% CI] across the three hold-out folds, i.e. the data portions used, in turn, as validation set in the three-fold cross-validation setting.

| | | Basal | Luminal | Indifferent |
|---|---|---|---|---|
| Repetition 1 | Precision | 0.43 [0.28-0.58] | 0.59 [0.43-0.75] | 0.11 [0-0.43] |
| | Recall | 0.43 [0.12-0.74] | 0.74 [0.56-0.91] | 0.05 [0-0.15] |
| | F1-Score | 0.43 [0.2-0.66] | 0.65 [0.5-0.81] | 0.06 [0-0.21] |
| | AUROC | 0.7 [0.5-0.9] | 0.66 [0.58-0.74] | 0.43 [0.25-0.61] |
| | Accuracy | 0.48 [0.32-0.65] | | |
| Repetition 2 | Precision | 0.51[0.38-0.64] | 0.63 [0.44-0.82] | 0.35 [0.15-0.56] |
| | Recall | 0.57 [0.1-1] | 0.69 [0.57-0.8] | 0.24 [0.05-0.43] |
| | F1-Score | 0.54 [0.24-0.83] | 0.65 [0.59-0.71] | 0.29 [0.1-0.48] |
| | AUROC | 0.75 [0.56-0.94] | 0.71 [0.44-0.99] | 0.58 [0.27-0.88] |
| | Accuracy | 0.55 [0.45-0.65] | | |
| Repetition 3 | Precision | 0.51 [0.37-0.64] | 0.55 [0.5-0.6] | 0.33 [0-0.7] |
| | Recall | 0.55 [0.45-0.65] | 0.59 [0.02-1] | 0.22 [0-0.57] |
| | F1-Score | 0.52 [0.46-0.59] | 0.55 [0.23-0.88] | 0.24 [0.06-0.41] |
| | AUROC | 0.77 [0.67-0.86] | 0.66 [0.62-0.71] | 0.55 [0.45-0.65] |
| | Accuracy | 0.48 [0.3-0.67] | | |

AUROC: area under the receiver operating characteristic curve; CI: confidence interval.

**Table S3. Performance metrics of the deep-learning classifier in the prediction of the luminal and basal protein-based subtypes for the three repetitions.** For each repetition a measure of accuracy, AUROC, sensitivity, specificity, precision, and F1-Score is reported for the basal subtype (here taken as the 'positive' class) as mean [95% CI] across the three hold-out folds, i.e. the data portions used, in turn, as validation set in the three-fold cross-validation setting.

| | Repetition 1 | Repetition 2 | Repetition 3 |
|---|---|---|---|
| Accuracy | 0.75 [0.52-0.99] | 0.79 [0.75-0.84] | 0.75 [0.48-1] |
| AUROC | 0.83 [0.67-0.99] | 0.8 [0.62-0.99] | 0.81 [0.65-0.96] |
| Sensitivity (Recall) | 0.67 [0.46-0.87] | 0.6 [0.49-0.7] | 0.6 [0.32-0.87] |
| Specificity | 0.8 [0.42-1] | 0.9 [0.84-0.96] | 0.83 [0.55-1] |
| Precision | 0.67 [0.25-1] | 0.76 [0.67-0.85] | 0.66 [0.2-1] |
| F1-Score | 0.66 [0.42-0.9] | 0.67 [0.6-0.73] | 0.62 [0.27-0.98] |

AUROC: area under the receiver operating characteristic curve; CI: confidence interval.

**Table S4. High-confidence predicted luminal and basal slides (see separate Excel file).** The table lists all slides predicted luminal (sheet 1) or basal (sheet 2) with a prediction score higher or equal than 0.7. For each slide the true label (column 'Subtype'), i.e. the label assigned by hierarchical clustering analysis, the prediction score for the basal (column 'PredScore_Basal') and luminal (column 'PredScore_Luminal') classes, the histopathological characterization (columns E-I), as well as the H-score associated with the six markers' set, are reported. High-confidence predicted luminal and basal slides are sorted by descending values of PredScore_Luminal and PredScore_Basal respectively.

**Table S5. Low-confidence predicted slides (see separate Excel file).** The table lists all slides for which the prediction score for the basal class was in the range [0.4-0.6]. For each slide the true label (column 'Subtype'), i.e. the label assigned by hierarchical clustering analysis, the prediction score for the basal (column 'PredScore_Basal') and luminal (column 'PredScore_Luminal') classes, the histopathological characterization (columns E-I), as well as the H-score associated with the six markers' set, are reported.
